# Analysis of Agro‐Environmental and Geo‐Climatic Factors Influencing Buruli Ulcer in the Kimpese Health Zone in Kongo Central, Democratic Republic of Congo

**DOI:** 10.1002/puh2.70111

**Published:** 2025-08-12

**Authors:** Jojo Mazama Sukami, Innocent Mufungizi, Julien Bompeta Lombo, Aymar Akilimali, Holenu Mangenda Holy

**Affiliations:** ^1^ Laboratory of Space Geodesy Astronomy and Geophysics Geographic Institute of Congo (GIC) Kinshasa DR Congo; ^2^ Faculty of Sciences and Technologies University of Kinshasa Kinshasa DR Congo; ^3^ Department of Geo‐Topography Scientific Directorate Geographic Institute of Congo Kinshasa DR Congo; ^4^ Pedology and Geochemistry Laboratory University of Kinshasa Kinshasa DR Congo; ^5^ Department of Research Medical Research Circle (MedReC) Bukavu DR Congo; ^6^ The Marine Biological Association (MBA) Plymouth UK

**Keywords:** Buruli ulcer | infectious disease | MBASU | *Mycobacterium ulcerans*

## Abstract

**Background:**

Infectious diseases are the most common causes of death and disability in the Democratic Republic of Congo (DRC). Among the wide variety of these infectious diseases, Buruli ulcer (BU), commonly known as MBASU, is one of the most aggressive. According to community beliefs, this disease is associated with mysticism and is treated traditionally.

**Aims and Methods:**

The overall objective of this study was to conduct a geo‐environmental and geo‐climatic analysis of the factors influencing the spread of BU in the Kimpese health zone. To achieve this, a field visit allowed us to collect certain data, including the type of activity of patients, the type of crops grown, the month of onset of the disease, and environmental factors. Thus, through geographic analysis, we were able to situate the disease in its environment.

**Results:**

Of a total of 35 people infected with *Mycobacterium ulcerans*, 51.4% were female and 48.6% were male. Overall, 45.7% of the patients were under 15 years of age. Agriculture is the activity engaged in by 34% of the infected people and constitutes a risk factor for the population. Overall, 82.9% of the people surveyed cultivate rice; 88.6% cultivate bananas; and 65.7% cultivate cassava. The number of cases was found to be higher during months of low rainfall. The lowest temperatures were observed in the most affected health areas.

**Conclusion:**

This study shows a correlation between agricultural activities and BU affecting populations who grow food crops near water sources; the same is true for the geo‐climatic factors taken into account.

## Graphical abstract

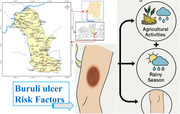

## 1 Introduction

In some tropical countries, populations are victims of both poverty and chronic or endemic diseases [1, 2], caught in a vicious cycle where the effects of disease and poverty combine and amplify each other [3]. Populations in developing countries are not always guaranteed basic healthcare [4]. It was with them in mind that the WHO launched a generous, but still very theoretical, slogan in the early 1980s: “Health for all by the year 2000” [5]. This has not been achieved. Developed countries are therefore becoming increasingly aware of this danger and are developing measures to mitigate or even stem the potential damage caused by the disease by introducing health insurance. However, in developing countries, such as the Democratic Republic of Congo (DRC), which barely has the resources to implement rural development strategies that can provide rural services and amenities for a decent living environment to the population [6, 7, 8], the population is left in uncontrolled rural exodus, leading to public health challenges [9]. Indeed, “Despite the scientific and medical advances of the 20th century, science and human knowledge are still faced with diseases that remain enigmas” [10, 11]. One of these diseases is “Buruli ulcer (BU),” and it is far from new [12, 13]. The disease was first recognized in Buruli Country in Uganda in 1897 [14]. The first observations were published in Australia in 1937 [15] and then in the former Belgian Congo in 1942 [15]. In the DRC, outbreaks have been described in almost all provinces. Before 1980, more than 500 cases of BU were reported. After a period of epidemiological silence of approximately 20 years, cases of BU have re‐emerged in areas where outbreaks were previously reported since the year 2000 [16]. A national survey organized in 2004 identified 487 probable cases of BU distributed in the provinces of Bas‐Congo, Bandundu, Katanga, Kinshasa, Maniema, and South Kivu [17]. In 2019, Idrissa et al. conducted a study on the phytochemistry of medicinal plants used against BU in Kongo Central by the Ntandu people. According to this study, *Aloe tenuifolia*, *Annona senegalensis*, *Brillantaisia owariensis*, *Vernonia amygdalina*, and *Strychnos icaja* are involved in the management of BU. Chemical screening revealed the presence, to varying degrees, of secondary metabolites that provided a scientific basis for the endogenous knowledge of the Ntandu [18]. The Kongo Central province contains one of the most important foci in the country, namely, the territory of Songololo, to which belongs the Kimpese health zone that is the study area of this work where practices remain predominantly traditional for the management of this public health problem.

This study aims to explore cases and potential factors in the climate and environment that may be important for BU in the Kimpese health zone, which is found in the Kongo Central province of the DRC. With traditional medicine being the major form of care because of limited access to up‐to‐date treatments, the community is lacking more modern and effective healthcare. It aims to explain how various environmental influences (including temperature, humidity, closeness to waterways, and soil type) may contribute to this neglected disease appearing and spreading in a region tied to witchcraft and where the usual treatment is traditional. We expect this research to highlight how the disease affects nature and aid in stopping this disease by informing local communities and starting targeted medical research.

## 2 Methodology

### 2.1 Description of the Study Area

**FIGURE 1:**
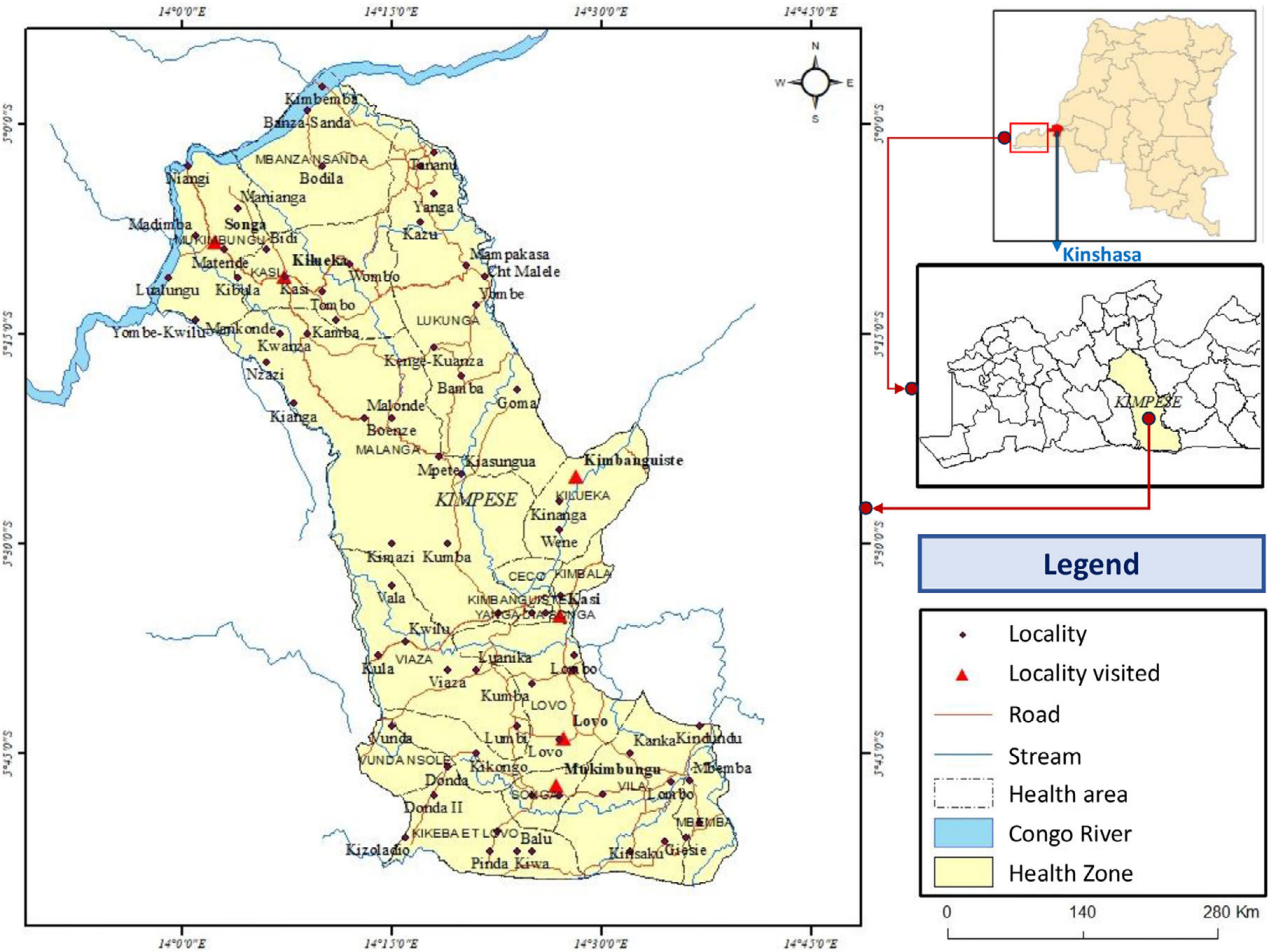
Location map of the study area: administrative map of the Kimpese health zone.

In 2024, the population of Kongo Central was 6.9 million. The national median age is approximately 16.9 years (men: ∼16.7 years, women: ∼17 years) according to 2024 estimates, with the overall national sex ratio very close to equality, with approximately 1 man per woman (or almost 100 men for 100 women) [19, 20]. The study was conducted in villages in the Kimpese health zone where confirmed cases of BU have been reported. The study area is located in the DRC in the Kongo Central province, more precisely, in the Songololo territory located between 13°30′ and 14°30′ E longitude and between 5°00′ and 5°50′ S latitude. The Kimpese health zone is one of the two zones that make up the Songololo Territory, namely, Kimpese and Nsona‐Mpangu (Figure 1).

### 2.2 Materials and Methods

#### 2.2.1 Materials

The material used for this study includes satellite images (Landsat) used including that of the Landsat 8 satellite with a spatial resolution of 30 m taken in the city of Kinshasa in June 2010 for the establishment of temperature and land use maps but also software, including the mapping and spatial analysis software Arc Gis 10.0 and Qgis 2.18, used in this work to analyze and process the data (digitization, creation of a geographic database, and development of maps); EpiData version 3.1 (EpiData Association, Odense, Denmark): for entering the survey data; Google Earth Pro that was used in the visualization of the location of satellite information in real time to verify the veracity of our downloaded satellite images; was used for data entry, and SPSS Statistics version 26.0 (IBM Corp., Armonk, NY, USA): for processing and statistical analysis of data.

#### 2.2.2 Methods

Two main methods were employed in this study: a statistical and a descriptive geographical. The statistical method was used to analyze and compare quantitative data collected from the field with existing information in order to identify patterns and verify consistency. This approach enabled a structured interpretation of the data and supported empirical observations. The descriptive method, on the other hand, aimed to provide a detailed inventory of the socio‐environmental characteristics of the study area. Using an inductive approach, it helped identify regularities in the spatial organization of rural areas in the Kimpese health zone, contributing to a comprehensive geographical understanding of the local context and its relationship with BU occurrence.

- Field survey

The study was comprehensive and covered the health areas where confirmed cases of BU were reported during the period of 1 year, from 2022 to 2023. The official health surveillance system of Kimpese health zone was used to recognize confirmed cases of BU. The authors have granted access to this information by cooperation between the health authorities and local authorities. These instances were later confirmed as they visited the cases in the villages. The data were collected first hand within the communities through the use of well‐structured interviews and a standardized description form meant to collect the appropriate demographic, environmental, and socio‐economic data pertinent to each validated patient.

Of the 135 people suspected of having BU in the Kimpese health zone during the period 2022–2023, only 35 were confirmed, either by the Evangelical Medical Institute of Kimpese, the National Institute of Biomedical Research of Kinshasa, or the Institute of Tropical Medicine of Antwerp. The study then involved grouping these patients first by health area and then by village and finally, conducting field visits to the villages where these patients were reported in order to collect useful information for the case study.

- Data collection

A descriptive sheet per respondent was completed in order to be able to record the various socio‐economic‐demographic and environmental variables, in particular, the age and sex of the patient, the place of birth of the patient, the level of education of the patient, the sex of the head of household, the type of occupation of the patient, the proximity of watercourses, the type of vegetation in the village and its immediate environment, the presence of marshes and the nature of the soil in the village and on the edge of the nearby watercourse, the types of crops practiced by people with BU, and the marshy environment.

- Delimitation of the subject

This study is limited in time and space. It covers the period from 2022 to 2023, and its spatial focus is on the Kimpese health zone due to time and funding constraints. This is why the study focused on a descriptive cross‐sectional survey of villages where confirmed cases of BU were reported in different health areas of Kimpese. The work focused on studying the geographic, rather than environmental, factors associated with the occurrence of BU in the Kimpese health zone, as the disease is primarily found in hot and humid regions, generally in foci in marshy or poorly drained areas.

### 2.3 Limitations of the Study

This study has several limitations that are taken into account when interpreting the results. First, the sample size remains relatively small, with only 35 confirmed cases of BU over the study period, which limits the statistical scope of the analyses and the generalizability of the results. Second, the data collection period, although recent, is partly based on cases confirmed between 2022 and 2023, introducing a temporal bias that may affect the accuracy of environmental and climatic correlations. Furthermore, the study focused solely on the Kimpese health zone for logistical and financial reasons, which prevents a broader assessment of the phenomenon in other endemic areas of the province or the country. Finally, although geospatial tools were used, some environmental data, such as water quality and precise soil composition, could not be collected directly in the field, limiting the depth of the environmental analysis.

## 3 Results and Discussion

### 3.1 Analysis of Variables Influencing the Observed Cases

As reported in Table 1, among all 35 participants, there were similar numbers of female (51.4%) and male participants (48.6% male), and many participants were children under 15 years of age (45.7%). This observation is explained by the fact that in rural areas, children and women play a significant role in the economic development of the community and in all the activities that contribute to the progress of the family, including fetching water, fetching bundles of wood, going to the market, and going to the fields. All these activities make them more vulnerable to this disease [21]. According to Ngoma, women are the people who work the most and almost without rest, day and night, in the city as well as in the village to maintain the family [22]. The authors Buntine et al. noticed that BU is more severe in poor populations in remote rural areas [23]. In more than 50% of cases, the disease affects children under 15 years of age [24, 25].

**TABLE 1:** Social and demographic variables.

Variables	Classification	Number	%
Sex	Female	18	51.43
	Male	17	48.57
	Total	35	100
Age group	0–15 years old	16	45.71
	16–49 years old	15	42.86
	>49 years old	4	11.43
	Total	35	100
Educational level	Preschool	3	8.57
	Primary	18	51.43
	Secondary/Vocational	10	28.57
	No level of education	4	11.43
	Total	35	100

In the context of this study, the link between age group and agricultural practices reflects both active involvement and passive exposure. Children under 15 years of age, who represent 45.7% of the study participants, are not merely bystanders but frequently engage in daily subsistence activities alongside adults. In rural communities, children often accompany their parents, particularly their mothers, to the fields, water points, and markets, and they actively participate in various agricultural tasks appropriate to their age. Such activities can be watering crops, assisting in planting and gathering, or merely being in the agricultural setting. Consequently, their exposure to BU is direct by way of direct contact with the hydro‐telluric environment as well as indirect via exposure during accompaniment of the relatives. Such a cross exposure could be a possible explanation as to why the prevalence of BU invokes kids and women, as seen in our data and confirmed by other research studies.

In rural environments such as the Kimpese health zone, children are frequently exposed to specific risk behaviors that increase their vulnerability to BU. These behaviors include swimming or bathing in stagnant or slow‐moving water bodies, playing barefoot near marshes or rice fields, and accompanying parents to the fields where they may come into contact with contaminated water and soil. Moreover, children can be taken to help with the simplest work in a farm like weeding or irrigation, especially during rice and bananas plantation, where a lot of water has to be used. These sequential and extended exposures to water can perhaps cause transcutaneous infection with *Mycobacterium ulcerans* aggravated by trivial skin trauma occurring in the course of recreation or work. Consequently, the daily activities of children automatically cause them to be more likely to be infected by BU in endemic regions.

Research carried out by Aubry and Gaüzere in 2021 showed that BU is a neglected tropical disease in rural areas, primarily affecting children under the age of 15, and an emerging public health threat in many humid intertropical rural regions [15]. Table 2 presents the various daily activities carried out by the participants, closely linked to the observations of environmental and occupational exposure mentioned above. These practices are particularly common among women and children and contribute to explaining their increased vulnerability to BU, as observed in our results.

**TABLE 2:** Types of profession of the surveyed populations.

Variable	Classification	Number	%
Type of profession	Unoccupied	21	59.99
	Agriculture	12	34.29
	Hunting	1	2.86
	Seller	1	2.86
	Total	35	100

Aside from those without occupations, agriculture is the activity engaged in by 12 of 35 (34.3%) of infected individuals. This study took place in a rural setting where agriculture is the main activity [26]. It is difficult to find a single person who does not practice agriculture, regardless of the type of activity they engage in. According to Nau, activities near a water source, such as agriculture, constitute a risk factor. BU is a disease linked to the aquatic ecosystem [27]. Human transmission is probably direct, transcutaneous, from the hydro‐telluric reservoir, and the rural population runs too great a risk of contamination by hydro‐telluric environments when visiting water points [28].

The results in Table 2 concern not only those who practice agriculture but also their children who accompany them. The results of the study conducted by Brou show that rice crops and, to a lesser extent, banana crops represent a high risk for the population because they require constant humidity and an intense supply of water [29]. This is unlike cassava, corn, and peanut crops, which do not require irrigation.

However, it should be noted that cassava is grown in marshes or near slow‐flowing rivers. Following the study by the authors Asiedu et al., BU is frequently observed in the immediate vicinity of stagnant or slow‐flowing water tables (slow‐flowing rivers, ponds, marshes, ponds, and lakes) [30]. Table 3 indicates that 82.9% of the people surveyed cultivate rice, 88.6% bananas, and 65.7% cassava. Consequently, BU would be linked to the presence of these types of crops because they require partial immersion of the farmers, and this permanent contact with water could thus be its cause.

**TABLE 3:** Types of crops grown by the people surveyed.

Variable	Classification	Number			%		
		No	Yes	Total	No	Yes	Total
Types of culture	Cassava	12	23	35	34.3	65.7	100
	Peanut	9	26	35	25.7	74.3	100
	Bean	7	28	35	20	80	100
	Pineapple	10	25	35	28.57	71.43	100
	But	6	29	35	17.1	82.9	100
	Rice	6	29	35	17.1	82.9	100
	Bananas	4	31	35	11.4	88.6	100
	Tomato	4	31	35	11.4	88.6	100
	Soy	7	28	35	20	80	100
	Onion	4	31	35	11.4	88.6	100

### 3.2 Bioclimatic Analysis

MacCallum and Tolhurst have shown that in many regions, *M. ulcerans* infections only appeared after significant ecological disturbances [31]. However, thanks to data collected and discoveries in molecular biology, the sources of *M. ulcerans* are becoming more clearly understood [32, 33, 34]. As several major endemic foci are found in swampy areas of tropical and subtropical countries, environmental factors must play a key role in the survival of the etiological agent [35, 36]. Epidemic foci have emerged following flooding, migration, and human‐made changes to the topography, such as dikes or recreational areas. Deforestation and increased agricultural activities may also have contributed significantly to the marked increase in the incidence of *M. ulcerans* infections observed recently, particularly in West Africa where the disease is emerging rapidly [37, 38, 39].

**FIGURE 2:**
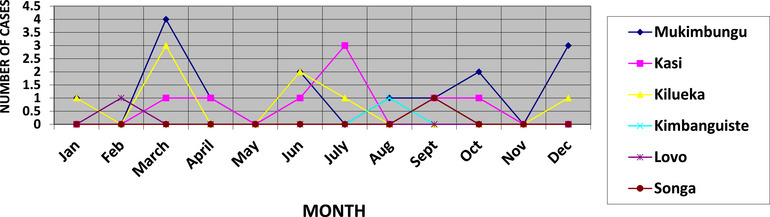
Monthly progression of confirmed BU cases by health area in the Kimpese health zone (2022–2023). The figure shows recurrent peaks that appear to follow specific seasonal patterns. These peaks follow environmental exposure, potentially linked to variations in rainfall, agricultural activities, or land use near water sources. Although the figure suggests a temporal clustering of cases, further correlation with temperature and land use data such as those presented in Figures 3 and 4 is important to more clearly demonstrate the strength of these associations.

**FIGURE 3:**
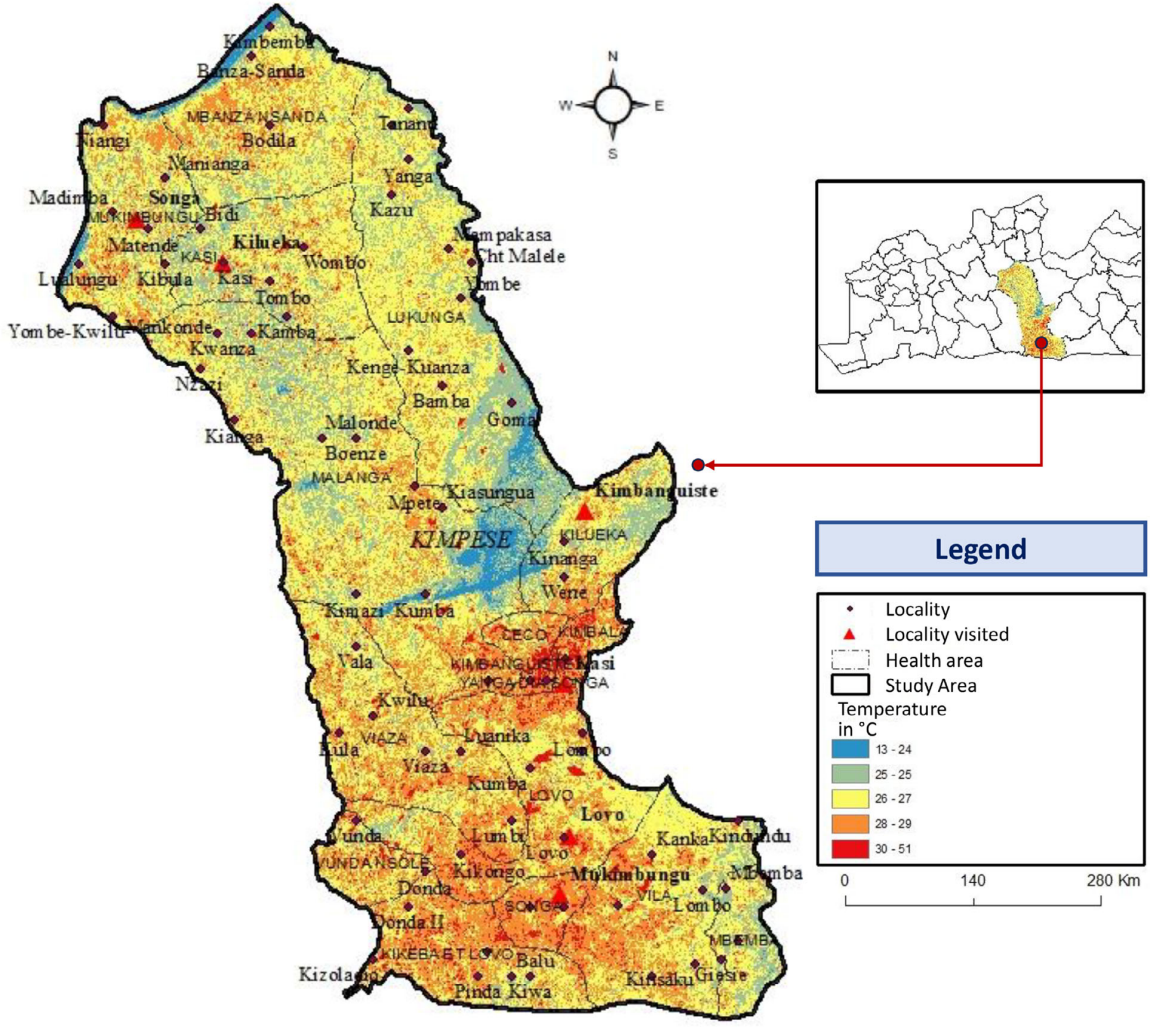
Temperature map of the Kimpese health zone.

As shown in Figure 2, we observe that the number of cases is higher during the months of rainfall deficit, including March and July. February to April and May to August correspond to the periods that followed those of heavy rains, therefore the growing season. In addition, it is also important to know that in rural areas, seasonal variations seem more linked to changes in population activities according to the seasons than to climatic conditions. Figure 3 presents the meteorological situation in the Kimpese health zone.

Vincent showed through a study on epidemiology and human genetics at the BU that several epidemics have been attributed to exceptional weather conditions: floods in Uganda in 1964 and floods in Papua in 1951 [40]. Figure 3 clearly shows that the lowest temperatures are recorded in the most affected health areas (Mukibungu, Kasi, and Kilueka). Several publications cited above stipulate that BU is endemic in all tropical and subtropical regions. The spread of the disease is believed to be linked to environmental changes caused by human activities (agriculture, population displacement). Environmental (climatic) conditions are crucial in the spread of tropical infections, with etiological agents (insect vectors and parasites) preferring warm and umid areas. Although BU is traditionally associated with tropical and subtropical regions in developing countries where poor sanitation and environmental exposure contribute to its spread, recent increases in incidence in temperate, high‐income settings such as Australia suggest that other factors, including ecological and climatic changes, land use, and specific local reservoirs, may also play a critical role in disease emergence [41, 42, 43, 44]. The important factors in the growth and spread of this bacterium are essentially temperature and humidity. The Kimpese health zone clearly demonstrates the high temperature it accumulates. These temperatures are favorable to the growth of BU, which leads to the presence of cases of this disease in the Kimpese health zone, along with seasonal and interannual variations in the climate of the Kimpese health zone. High rates of the disease seem to be strongly associated with seasonal variations, as a consequence of which BU becomes prevalent during rainy season, presumably due to more intensive exposure to environment and suitable conditions of the bacterium spread.

**FIGURE 4:**
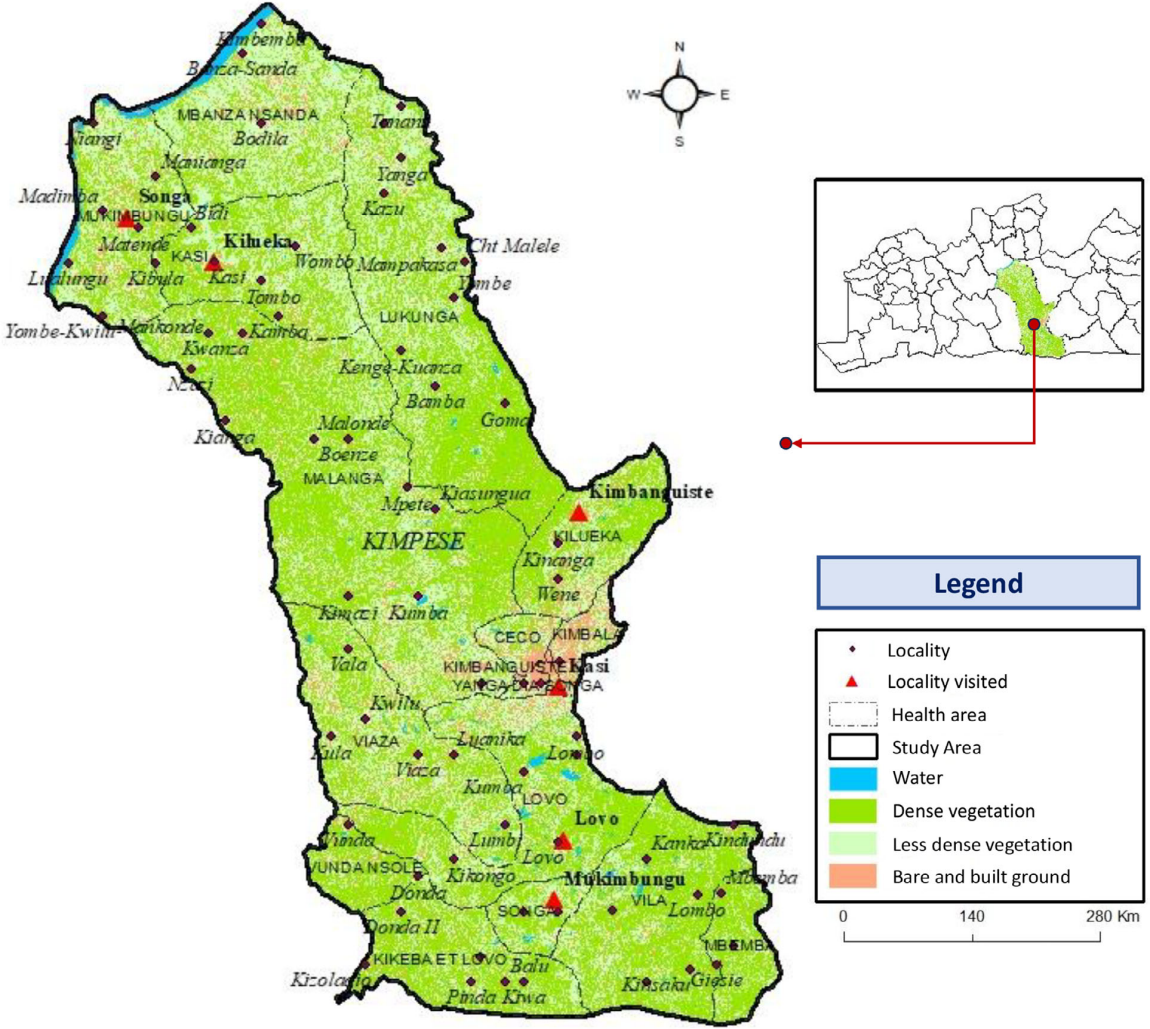
Land use map of the study area. This study investigates the link between Buruli ulcer (BU) and agro‐environmental and geo‐climatic factors in rural settings. By analyzing land use, water proximity, and rainfall patterns, it highlights key exposure risks and reinforces the need for targeted environmental surveillance and preventive measures in endemic areas to reduce BU transmission.

According to Figure 4, the environmental change factor could be implicated in the health areas of Mukibungu, Kasi, and Kilueka, which are the most affected by BU. These areas have an environment favorable to the development of agriculture. In particular, vegetation consisting of degrading open forest, gallery forests, wooded valleys, and shrub savannahs. Deforestation, increased agricultural activities and the creation of new residential areas could therefore have contributed significantly to the increase in the incidence of BU infections in these health areas.

## 4 Conclusion

According to the study, BU distribution in the Kimpese health zone is strongly influenced by the region's geo‐environment and geo‐climatic factors, which are very compelling. A link between farming rice, bananas, and cassava near water sources and the probability of getting infections from *M. ulcerans* clearly shows that the interaction between humans and their environment plays a key role in transmission. It is clear from these numbers that there are important social and behavioral factors behind the issue, which should be studied more deeply. All in all, these discoveries prove that it is crucial for public health to manage the environment, spread relevant messages, and strengthen surveillance to protect rural communities from BU.

## Author Contributions

All authors participated in the conception of the study, contributed to the drafting of the manuscript, and approved the final version. All authors have read and approved the final version of the manuscript. The corresponding author had full access to all of the data in this study and takes complete responsibility for the integrity of the data and the accuracy of the data analysis.

## Ethics Statement

The study protocol was reviewed and approved by the Ethics Review Board of the Department of Research of the Medical Research Circle (MedReC) in DR Congo following the relevant approval letters, Ref/00026/02/LO.DR‐MedReC/2025 and the approval letter: MedReC/DR/0014/2025, respectively. The study design incorporated the separation of patient identification data by confidentiality codes to maintain anonymity and protect the privacy of the participants.

## Consent

Informed consent was obtained from all participants involved in the study. This study was performed in accordance with the ethics standards as laid in the 1964 Declaration of Helsinki and its later amendments or comparable ethics standards.

## Conflicts of Interest

The authors declare no conflicts of interest.

## Transparency Statement

The manuscript guarantor, Innocent MUFUNGIZI, affirms on behalf of all authors that this manuscript is an honest, accurate, and transparent account of the study being reported; that no important aspects of the study have been omitted; and that any discrepancies from the study as planned (and, if relevant, registered) have been explained.

## Data Availability

The data used for this work will be made available upon reasonable request from the corresponding author.
